# Small steps and giant leaps

**DOI:** 10.7554/eLife.08366

**Published:** 2015-06-03

**Authors:** Bram Prevo, Erwin JG Peterman

**Affiliations:** Department of Physics and Astronomy, and LaserLaB Amsterdam, VU University Amsterdam, Amsterdam, The Netherlandsb.prevo@vu.nl; Department of Physics and Astronomy, and LaserLaB Amsterdam, VU University Amsterdam, Amsterdam, The Netherlandse.j.g.peterman@vu.nl

**Keywords:** optical trap, molecular motor, single molecule, *E. coli*, Human

## Abstract

A study of kinesin-1 has shed new light on how motor proteins are able to move along microtubules inside cells.

**Related research article** Andreasson JO, Milic B, Chen G-Y, Guydosh NR, Hancock WO, Block SM. 2015. Examining kinesin processivity within a general gating framework. *eLife* **4**:e07403. doi: 10.7554/eLife.07403

**Image** Kinesin-1 can take over one hundred steps along a microtubule before falling off

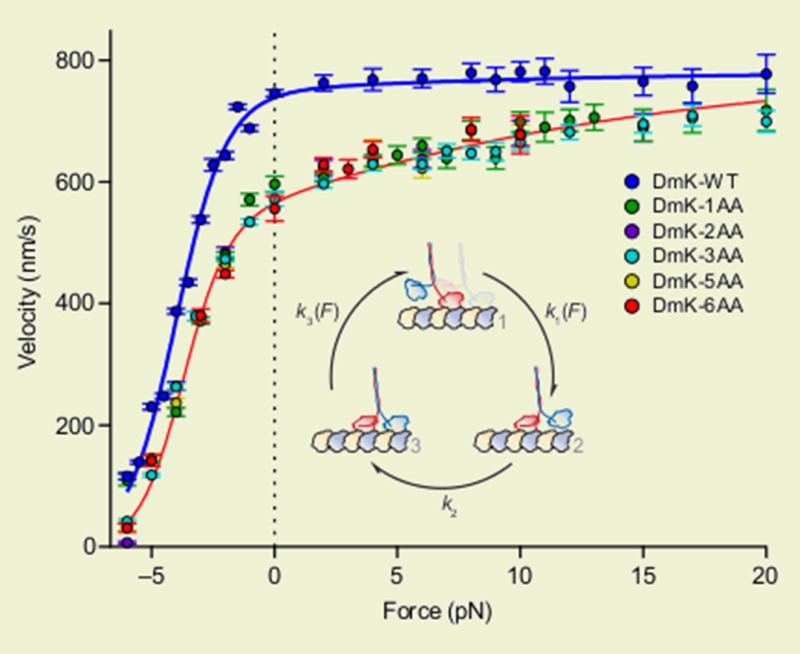

The world inside a cell differs dramatically from the world around us: inertia plays no role on the nanometer scale, while thermal fluctuations govern the way things move. Kinesins are motor proteins that are perfectly adapted to work in these conditions (Hirokawa et al., 2009). They move along filaments called microtubules to transport cargo through the cell by harnessing the energy obtained from the hydrolysis of ATP.

After binding to a microtubule, a single kinesin protein can take more than one hundred steps along it before detaching, and many aspects of this characteristic—which is called processivity—are not fully understood (Block et al., 1990; Vale et al., 1996). Now, in *eLife*, Steven Block from Stanford University, William Hancock from Pennsylvania State University and colleagues—including Johan Andreasson and Bojan Milic as joint first authors—provide new insights into how a kinesin called kinesin-1 steps along microtubules (Andreasson et al., 2015).

Kinesin-1 is a dimer that consists of two identical motor domains (heads), which both contain an ATP-binding pocket and a microtubule-binding domain (Vale and Milligan, 2000). Specialized regions called the neck linkers connect the heads to a stalk that is responsible for the formation of the dimer and for binding to the cargo. Kinesin-1 moves along the microtubule in ‘steps’ of 8.2 nm (Yildiz et al., 2004). A single step requires the heads to undergo a series of tightly coupled structural and enzymatic transitions, called the mechanochemical cycle (Hua et al., 1997; Schnitzer and Block, 1997).

For kinesin-1 to step along the microtubule, it is crucial that at least one of the heads is tightly bound to the microtubule at any time (Hancock and Howard, 1998). This means, for example, that the head that is attached to the microtubule should not unbind until the other head finds and securely docks to the next available binding site further along the microtubule, much like the way a tightrope walker must always have one foot in contact with the rope (Figure 1).

**Figure 1.**
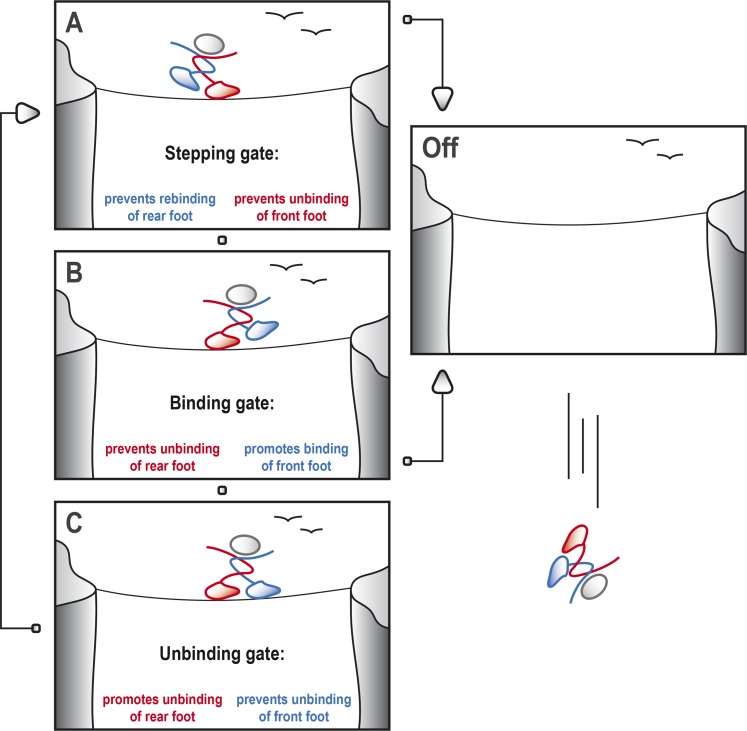
Kinesin-1 cycles through three gates to step along a microtubule. Kinesin-1 moves along a microtubule in a similar way to how a person would walk successfully along a tightrope. (**A**) When the tightrope walker (moving from left to right) has his/her front foot (red) in contact with the rope, the ‘stepping gate’ holds the red foot on the rope and keeps the rear foot (blue) away from the rope. (**B**) The blue foot moves in front of the red foot and the ‘binding gate’ allows the blue foot to contact the rope while preventing the red foot from coming away. (**C**) Now that the blue foot is in contact with the rope, the ‘unbinding gate’ allows the red (rear) foot to leave the rope while holding the blue foot in place. Cycling through these gates will ensure that at least one of the feet is tightly connected to the rope at all times, which allows the tightrope walker to cross the canyon safely. However, if any of these gates should fail, both feet may lose contact with the rope, resulting in a disastrous fall. This figure is based on the general gating framework for kinesin-1 by Andreasson et al. (2015).

When we are walking, our brain and nervous system are in control and coordinate our movements. Kinesin, however, does not have a central control system, so how does it coordinate the movements of the two heads? The only way a head can know the relative location of its partner, and whether it is bound to the microtubule, is via mechanical strain, which is relayed from one head to the other via the neck linkers. This allows the heads to communicate in order to control their stepping, which is also called ‘gating’ (Block, 2007).

Andreasson, Milic et al. have identified three gates—referred to as the ‘stepping’, ‘binding’ and ‘unbinding’ gates—that are crucial parts of the stepping cycle for kinesin and other dimeric motor proteins (Figure 1). At each gate there is a mechanochemical transition that results in changes to the conformation of the neck linkers, the strength of the binding between the heads and the microtubule, and the kinetics of ATP hydrolysis. To explore the role of the gates in processive movement, Andreasson, Milic et al. compared wild type kinesin-1 motors and mutant kinesin-1 motors with longer neck linkers in a series of experiments: in particular, they measured how the speed of the motors, the run length (that is, the total distance moved) and the rate at which the heads detached from the microtubules depended on the force between the two heads.

The experiments revealed that increasing the length of the neck linker only moderately affected the stepping gate, resulting in some rebinding of the rear head. However, the binding gate was more substantially affected, as was evident from the shorter run lengths of motors with a neck linker that was only one amino acid longer than normal.

Remarkably, the unbinding gate—which acts when both heads are bound to the microtubule, and might therefore be expected to be very sensitive to any lowering of the force between the heads—did not affect processivity. The motors with longer neck linkers moved at a slower speed, but this could be partially restored by applying a force in the direction of motion. Andreasson, Milic et al. thus propose that, at the unbinding gate, it is not the tension between the heads that coordinates the mechanochemistry of the heads: rather, it is the spatial orientation of the neck linkers (in particular, the spatial orientation of the neck linker belonging to the front head) that is responsible for coordination.

Taken together, these results shed important new light on the gating mechanisms that ensure that kinesin-1 can move along the microtubule, including how these mechanisms depend upon the neck linkers and tension between the heads. It appears that the length of the neck linker is optimized for speed and processivity. In the future, it would be interesting to find out whether there are regulatory mechanisms in the cell that specifically affect the gates.

The general gating framework and the experimental and modeling approach pioneered by Andreasson, Milic, Block, Hancock and colleagues can be applied to other kinesins, but also to other motor proteins, such as myosin and dynein. It will be an exciting challenge to understand how the mechanisms and structures of motor proteins are optimized for other characteristics of movement, such as the generation of force, or their ability to work together in teams.
